# Oral hygiene and oral microbiota in children and young people with neurological impairment and oropharyngeal dysphagia

**DOI:** 10.1038/s41598-021-97425-x

**Published:** 2021-09-10

**Authors:** Luiz Fernando Fregatto, Isabela Bazzo Costa, Daniel De Bortoli Teixeira, Janaina Costa Marangon Duarte, Aline Maria Noli Mascarin, Salum Bueno da Silveira Junior, Bianca Eduarda Baptistella Mesquita Serva, Roberta Gonçalves da Silva, Francisco Agostinho Junior, Paula Cristina Cola

**Affiliations:** 1 University of Marilia (UNIMAR), Av. Hygino Muzzi Filho, 1001, Mirante, Marília, São Paulo Cep: 17.525-902 Brazil; 2 Graduate of Speech, Language and Hearing Sciences Department, Dysphagia Research Rehabilitation Center, São Paulo State University-UNESP, Marília, SP Brazil

**Keywords:** Molecular biology, Neuroscience

## Abstract

This study compared the oral hygiene and oral microbiota in children and young people with neurological impairment and oropharyngeal dysphagia with and without gastrostomy. Forty children and young people participated in this study: 19 females and 21 males, aged 2 to 22 years (mean age 8.6 years). Participants were divided into two groups: group I (GI = 20) with gastrostomy and group II (GII = 20) without gastrostomy (with oral feeding). Oral hygiene was assessed using the Simplified Oral Hygiene Index (SOHI). Analysis of two bacteria, *Streptococcus mutans* and *Streptococcus sobrinus*, was performed by collecting saliva using an oral swab, then mRNA expression was evaluated using the polymerase chain reaction (PCR) technique. The oral hygiene index had a general median of 2.2, and the two groups were statistically different (Group I: median 2.9 and Group II: median 2.0) (p = 0.01751). Bacterial analysis indicated 13 individuals with *S. mutans* and none with *S. sobrinus*. Of the 13 individuals with *S. mutans*, 6 were from Group I and 7 from Group II. Those with gastrostomy had worse oral hygiene, and both groups harbored the bacterium *S. mutans*.

## Introduction

Children and young people with neurological impairment have a high prevalence of oropharyngeal dysphagia that can reach 99%, depending on the assessment method and the population studied. A study involving children diagnosed with severe cerebral palsy and cognitive impairment found a 99% prevalence of dysphagia. In another study, the authors found a prevalence of dysphagia in 83% of children with cerebral palsy. Both studies used clinical evaluation methods. Another study, using a clinical and instrumental method to evaluate children with quadriplastic cerebral palsy, found changes in the oral and pharyngeal phases of swallowing, with rates that reached up to 93.7% of research participants^1–3^.

Children and young people with neurological impairment also have a greater predisposition to develop bacterial plaque due to the difficulty of maintaining oral hygiene themselves or to inability on the part of those responsible for monitoring this care. In addition, dysphagia itself is a factor that contributes to the formation of dental plaques or stones, since it has a close relationship with oral hygiene, both in children and young people with gastrostomy, as well as those with oral feeding^4,5^.

There are few studies in the literature that analyze oral hygiene in children with neurological impairment using an alternative route of food intake such as gastrostomy, and the available studies are not recent. So far, the evidence suggests that there is a greater decline in oral hygiene in children using gastrostomy; this can be explained by the inefficient oral motor control seen in severely compromised children, which has a negative influence on oral conditions, particularly oral hygiene^6^. In another study, the authors examined the effect of food consumption on subgingival bacteria levels in children with gastrostomy and healthy children, finding that the two groups exhibited similar subgingival bacteria^7^.

The presence of bacteria in the oral cavity has frequently been analyzed, but there are few studies involving children and young people with neurological impairment and the use of gastrostomy. It is clear that children with gastrostomy have significantly more plaques and calculus on their teeth, and more Haemophilus influenzae, with additional tendencies to more gram-negative, Pseudomonas, and Streptococcus pneumonia bacteria. For this reason, children with gastrostomy are more associated with aspiration pneumonia than children without gastrostomy^8^.

Among the most studied bacteria in relation to the presence of plaque and dental calculus is *Streptococcus mutans*. In the population of children with neurological impairment and use of gastrostomy, one study compared children with and without the use of gastrostomy with healthy children, and the results pointed to a lower prevalence of *S. mutans* in the oral cavity of children using gastrostomy^9^. A more recent study analyzed periodontal status in children with cerebral palsy; the authors concluded that the state of oral hygiene and the severity of periodontitis worsen as the stiffness and movement of muscle tone increases in individuals with cerebral palsy^10^.

*Streptococcus mutans* has been identified as the main bacterium in the etiology of caries, and *Streptococcus sobrinus* has a cariogenic potential in humans^11^. An altered state of oral health negatively affects general health and quality of life, contributing to increased rates of dental caries and periodontal disease. Difficulty in accessing dental care and lack of awareness of caregivers are real problems for the care of this population^12^.

The hypothesis of this study is that children and young people with neurological impairment with oropharyngeal dysphagia and using alternative food (gastrostomy) present more changes in oral hygiene and oral microbiota than children and young adults with oral feeding.

Considering the scarcity of studies on children with neurological involvement and use of gastrostomy, this study aims to compare oral hygiene and oral microbiota in children and young people with neurological involvement and oropharyngeal dysphagia with and without the use of gastrostomy.

## Methods

Forty children and young people participated in this study: 19 males and 21 females, aged 2 to 22 years old (mean age 8.6 years). All had neurological impairment (cerebral palsy and genetic syndrome) and neurogenic oropharyngeal dysphagia confirmed in their medical records. Participants were divided into two groups: Group I (GI, n = 20), composed of children and young people using gastrostomy, and Group II (GII, n = 20), composed of children and young people with oral feeding. Exclusion criteria were : children and young people with physical conditions that prevented the collection of clinical specimens, with an unstable general clinical picture, with the absence or incomplete eruption of dental elements number 11, 31, 16, 26, 36, and 46 (permanent dentition) or 51,71, 55, 65, 75, and 85 (primary dentition) necessary for evaluation and continuous antibiotic use.

The research project was approved by the Research Ethics Committee of the institution under the number 4.391.196. All those responsible for the individuals included in the study protocol gave informed consent for participation. The selection of children and young people occurred according to their presence in outpatient care and previous analysis of medical records confirming the diagnosis of oropharyngeal dysphagia. They were invited to participate in the research and received explanations about the objectives and the method of collection. Such children and young people are followed up at the Specialties Clinic of Hospital XXX.

Assessment of oral hygiene and saliva collection were always performed by the same two professionals, one from the dentistry area and the other from the nursing area, both trained and experienced in the field. The professionals receive training to complete the oral hygiene assessment form and to collect saliva. The training consisted of images with visual support provided by the scale itself and prior analysis for consensus. The child or young person was positioned in the Kavo dental chair, model Unik, or even in their own wheelchair, according to the positioning need. During evaluation and collection, sterile gloves, dental oral clinical mirror, dental explorer probe, disposable plastic sucker, triple syringe attached to the dental chair with an air and water jet, reflector light from the dental chair, and an oral swab were used.

### Simplified Oral Hygiene Index (SOHI)

**Figure 1:**
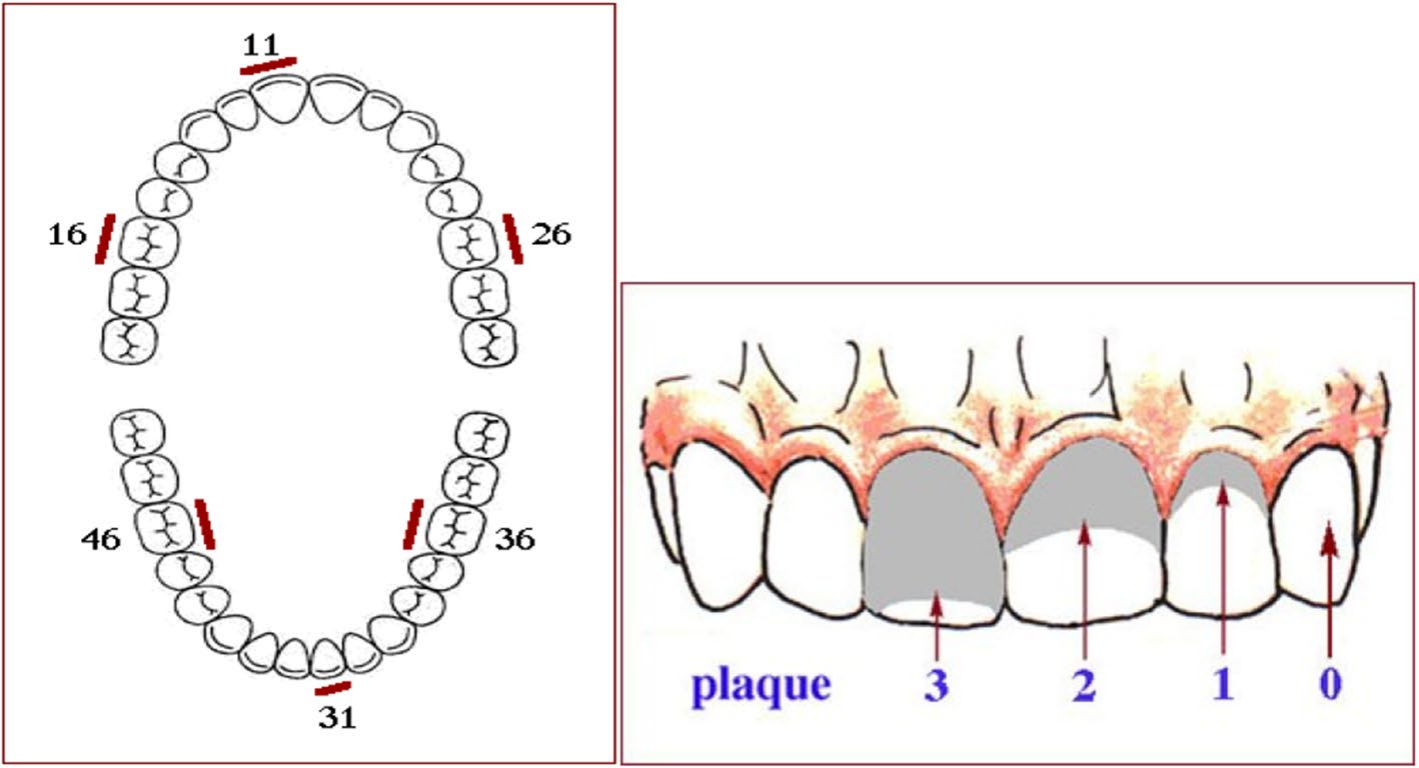
Malmo University oral healthy database.

In order to qualify oral hygiene, quantification of plaque deposit and dental calculus was performed in the entire sample population involved. Evaluation was carried out by the same professional specializing in the field of pediatric dentistry, with seven years of experience in serving this population. For this, the Simplified Oral Hygiene Index (SOHI) proposed by Greene and Vermillion^13^ was applied, in which the existence of a plaque or calculus was verified on the buccal surface of the number 11 tooth (upper right central incisor), 31 (lower left central incisor), 16 (upper right first molar) or 26 (upper left first molar), or on the lingual surface of tooth 36 (lower left first molar) or 46 (lower right first molar). In the absence of one tooth, we replaced it with another from the same group, used the same dental arch and the same quadrant; that is, we used the same dental arch and the same quadrant. Only fully erupted tooth were considered. In deciduous or mixed dentition, the buccal surface of tooth number 51 (upper right central incisor), 71 (lower left central incisor), 55 (upper right first molar), 65 (upper left first molar) was evaluated, as well as the lingual surface of tooth 75 (lower left first molar) and 85 (lower right first molar) (Fig. 1).

### Saliva collection

After cleaning the mouth with 100 mL of water, saliva was collected by scraping the inside of the cheeks with sterile swabs, making circular movements approximately 30 times. These swabs were cut and placed in 2 mL microtubes with gel inside. The collected samples were stored in a refrigerator at 4 °C for a period of up to seven days before the extraction of genomic DNA.

### Bacterial analysis

The saliva collected by oral swab allowed the investigation of two bacteria, Streptococcus mutans and Streptococcus sobrinus, through the expression of mRNA as measured by polymerase chain reaction (PCR). The analysis process is described below:

DNA was extracted with the commercial DNA isolation kit (Puregene, Gentra Systems, Minneapolis/EUA). In the microtubes containing the swabs, 300 µL of lysis solution was added. Then, 1.5 µL of proteinase K (20 mg/mL) and 100 µL of precipitation solution were added. Then, 300 µL of 100% isopropanol and 0.5 µL pf glycogen (20 mg/mL) were added, and the tubes were centrifuged at 1500 rpm for 3 min. The supernatant was discarded, and the tube was inverted onto absorbent paper. Then, 300 µL of 70% ethanol was used to wash the DNA. The tubes remained open 15 min for evaporation of residual ethanol, and the DNA was dissolved in 20 µL of DNA elution solution.

Extracted DNA samples were subjected to electrophoresis in 1.5% agarose gel in TBE (tris, boric acid and EDTA 0.001 M, pH 8.0) containing ethidium bromide at a concentration of 0.5 ug/mL of gel and observed in a Hoefer transluminator (model Macro-Vue UV-20) to check its integrity. The concentration of the DNA samples obtained were measured in a spectrophotometer (Ultrospec III, Pharmacia LKB Biochrom Ltd, Cambridge, England), at 260 nm. The 260/280 ratio equal to 1.8 was used to characterize the purity of the material. The samples were stored at 4 ºC until use.

In PCR, amplification of the constitutively expressed gene glyceraldehyde 3-phosphate dehydrogenase (GAPDH) was carried out as an internal control for the reactions to confirm whether the DNA extraction process was successful. The Invitrogen protocol was adopted: in a sterile tube was added 2.5 µL of 10 × PCR buffer, 0.75 µL of MgCl2 (50 mM), 0.5 µL of dNTP mix (10 mM), 1.0 µL of oligonucleotide primer forward (2 mM), 1.0 µl of oligonucleotide primer reverse (2 mM), 0.5 µL of DNA sample, 0.2 µL of Taq DNA polymerase (5 units/µL) and distilled and autoclaved water to complete the final volume of 25 µL. The reaction was performed on the Perkin Elmer GeneAmp PCR System 2400 thermal cycler, under the conditions of 94 °C for 45 s (denaturation), 60 °C for 45 s (annealing), 70 °C for 1 min (extension), and 70 °C for 15 min (final extension), for 25 cycles.

The FIREpol protocol was adopted for the amplification of *S. mutans* and *S. sobrinus* by PCR. In a sterile tube, 4 µL of 5 × Master Mix, 0.6 µL of forward primer oligonucleotide (10 µM), 0.6 µL for reverse primer oligonucleotide (10 µM), 1.0 µL of the DNA sample and distilled, autoclaved water was added to give the final volume of 20 µL. This reaction was also performed on the Perkin Elmer GeneAmp PCR System 2400 thermal cycler, under conditions of 95 °C for 30 s (denaturation), 59 °C for 30 s (annealing), 72 °C for 1 min (extension) and 72 °C for 5 min (final extension).

The sequences of the oligonucleotide primers for the amplification of GAPDH and specific bacteria are show in Table 1.

**Table 1:** Sequences of oligonucleotide primers used for the amplification of GAPDH and bacteria *Streptococcus mutans* and *Streptococcus sobrinus*.

Target gene	Sequence	Fragment
GAPDH	F 5ʹ TGT TCC AGT ATG ATT CCA CC 3ʹ850 bp	850 bp
	R 5ʹ TCC ACC ACC CTG TTG CTG 3ʹ	
*S. mutans*	F 5ʹ ACT ACA CTT TCG GGT GGC TTG G 3ʹ	517 bp
	R 5ʹ CAG TAT AAG CGC CAG TTT CAT C 3ʹ	
*S. sobrinus*	F 5ʹ GAT AAC TAC CTG ACA GCT GAC T 3ʹ	712 bp
	R 5ʹ AAG CTG CCT TAA GGT AAT CAC T C 3ʹ	

*bp* base pairs.

The amplified DNA samples were subjected to electrophoresis on 1.5% agarose gel in TBE containing ethidium bromide at a concentration of 1.0 µg/mL of gel and observed in a Hoefer transilluminator model Macro-Vue UV-20 to verify the expression of the analyzed genes.

### Statistical analysis

The data were previously analyzed for normality using the Kolmogorov–Smirnov test at 5% probability. Once the presence of the normal distribution of the analyzed variables was not confirmed, it was decided to use non-parametric statistics. Thus, the Mann–Whitney test was applied to compare groups in relation to oral hygiene, with the median and interquartile range (IQR) values being presented together for each group. For the analysis of bacteria, the chi-square test was used. For both tests, the level of 5% probability was used. All statistical analyses were conducted using the R software package.

### Ethical approval

All procedures performed in studies involving human participants were in accordance with the ethical standards of the institutional and with the 1964 Helsinki declaration and its later amendments or comparable ethical standards.

### IRB approval

The study was approved by by the Research Ethics Committee of the institution under the number 4.391.196. November 04, 2019. A non-opposed consent was obtained. The study was conducted according to the guidelines of the Declaration of Helsinki, and approved by the Institutional Review Board (or Ethics Committee) of Marilia University (protocol code 4.391.196).

### Consent for publication

A non-opposed consent was obtained for all participants.

### Informed consent

Informed consent was obtained from all those responsible for the individuals participants included in the study.

## Results

Table 2 shows that both groups had an oral hygiene index with values considered inadequate. In the comparison between GI and GII, it was observed that children and young people with gastrostomy had a more altered oral hygiene index, with a statistically significant difference in relation to GII (p = 0.01751). Table 2—Absolute and relative frequency (%) of individuals with the presence and absence of the bacteria Streptococcus mutans and Streptococcus sobrinus in groups I and II.

**Table 2:** Values of medians and interquartile range (IQR—first and third quartiles) of the oral hygiene index in relation to groups I and II and the total number of children and young people.

Groups	Median	IQR	p-value
GI (n = 20)	2.9	2.0 – 4.15	0.01751
GII (n = 20)	2.0	1.53 – 2.53	
TOTAL (n = 40)	2.2	1.6 – 3.08	

*GI* group with gastrostomy, *GII* Group without gastrostomy.

Table 3 indicates the presence of two bacteria, *S. mutans* and *S. sobrinus*, in the oral cavity of 37 children and young people (data from three patients was not considered due to technical error in the collection of the material). The presence of *S. mutans* can be observed in 13 (35.2%) children and young people, six (31.6%) GI and seven (38.9%) GII, without statistically significant difference (p = 0. 9037) between the two groups. *S. sobrinus* was not found in any of the members of either group.

**Table 3:** Absolute and relative frequency (%) of individuals with the presence and absence of the bacteria *Streptococcus mutans* and *Streptococcus sobrinus* in groups I and II.

PCR	SM			SS		
	G I (n = 19)	G II (n = 18)	Total (n = 37)	G I (n = 19)	G II (n = 18)	Total (n = 37)
Positive	6 (31.6%)	7 (38.9%)	13 (35.2%)	0 (0%)	0 (0%)	0 (0%)
Negative	13 (68.4%)	11 (61.1%)	24 (64.8%)	19 (100%)	18 (100%)	37 (100%)
p-value	0.9037					

*GI* group with gastrostomy, *GII* group without gastrostomy, *PCR* polymerase chain reaction, *SM* Streptococcus mutans, *SS* Streptococcus sobrinus.

## Discussion

The aim of this study was to analyze children and young people with oropharyngeal dysphagia with and without the use of the alternative route of feeding, as well as its relationship with oral hygiene and oral microbiota. The oral hygiene index was analyzed using a clinical protocol and verified that there were changes in oral hygiene. This finding is in agreement with the literature, which shows that children diagnosed with cerebral palsy are not able to take care of their oral health due to cognitive and motor changes, as well as their limited ability to communicate and dependence on their parents to recognize their pain and distress^14–18^.

The results, comparing children and young people with and without gastrostomy, showed that the oral hygiene index is worse in the group using gastrostomy. This finding is in agreement with data in the literature that indicate that the use of gastrostomy may be associated with changes in oral hygiene^19^. A study carried out with children using gastrostomy revealed that oral hygiene is more affected when compared to children who do not use gastrostomy. The authors concluded that children with gastrostomy, due to partial or total elimination of intraoral mechanical forces that occur during chewing, allow the deposition of pathogenic bacterial plaque and dental calculus, and this scenario has a negative influence on oral conditions, in particular in the oral hygiene index in individuals with gastrostomy^6^.

Another study that evaluated oral health conditions through salivary and microbiological parameters in individuals with cerebral palsy, with or without gastrostomy, showed a statistical difference between the groups regarding the plaque index, with the gastrostomy group presenting the highest indices of plaque^19^. Another study compared oral hygiene in children with special needs, with and without gastrostomy, in relation to the presence of plaque and dental calculus, and showed that children with gastrostomy had more plaques and calculi in relation to children without gastrostomy^8^.

The fact that the presence of plaques and dental calculi is greater in individuals with gastrostomy has been discussed in the literature. One explanation is that the pH of saliva in individuals using an alternative feeding route is more basic, due to the absence of food in the oral cavity. According to the literature, when the pH of the saliva is above 5.5, the fluid in the plate becomes supersaturated and tends to deposit mineral. Calculus formation probably occurs when the pH of the plaque remains above the critical level for a prolonged period, a situation that occurs in patients who are feeding alternatively (gastrostomy), and their plaque is not exposed to fermentable carbohydrates^9^.

Thus, individuals with gastrostomy have more basic saliva, and this can compromise the salivary components. Saliva is a complex mixture, formed mainly by secretions from the salivary glands, and it is essential for maintaining healthy oral tissues, as it lines the oral mucosa and protects against irritation, forms an ion reservoir for dental remineralization, aids in swallowing, and has antimicrobial action^20^.

In this study, the oral microbiota was also evaluated. The presence of two bacteria in the oral cavity, *S. mutans* and *S. sobrinus*, is associated with changes in oral hygiene as well as periodontal changes^21,22^. The results of this study showed that *S. mutans* was present in 35.2% of participants. *S. sobrinus* was absent in all children and young people. These results corroborate with the literature on *S. mutans*. *S. sobrinus*, which was not found, is associated with dental caries^21^, and evaluation of dental caries was not carried out in this study, though it should be investigated in future studies.

The most recent studies involving these two bacteria are aimed at children without neurological involvement and found that *S. mutans* and *S. sobrinus* are associated with the presence of caries. They also show that when identifying the presence of the two concomitant bacteria, the status of caries in early childhood is more serious, related to the greater consumption of soft drinks and sweets^21,23–25^.

Although most recent studies involve individuals without neurological impairment, there are studies in the literature related to the presence of these bacteria in individuals with neurological impairment, such as children with cerebral palsy. Such studies showed a higher prevalence of *S. mutans* in the oral cavity of children with neurological impairment when compared to children without neurological impairment. The presence of this bacterium is usually associated with a higher rate of plaque. Daily plaque removal is recommended for children with neurological impairment, as well as reduction in the amount and frequency of sugar ingestion, use of fluoride, and salivary stimulation^26^.

In this study, when comparing the presence of *S. mutans* in children and young people with gastrostomy with those without gastrostomy, there was no difference in the presence of the bacterium. This finding corroborates with the literature, as in a study carried out with 48 individuals divided into 3 groups: 15 individuals with cerebral palsy, 17 individuals with cerebral palsy and use of gastrostomy, and 16 individuals without neurological involvement. Caries index, plaque index, presence of *S. mutans*, and salivary buffering capacity were evaluated. The authors detected the presence of the bacterium *S. mutans* in the three groups without statistical difference^19^.

Studies from past decades have also shown that there is no difference in the presence of bacteria in the oral cavity, when comparing individuals using gastrostomy with healthy individuals without gastrostomy. This study evaluated the prevalence of subgingival bacteria levels in children using gastrostomy and in the control group with healthy children. The method used to analyze the bacteria was through PCR, as in this study. The authors concluded that both groups exhibited similar microbiological composition^7^.

Thus, in view of the results of this study, and based on scientific evidence, the importance of oral hygiene care in individuals with neurological impairment is highlighted. Periodontal diseases, in addition to impairing oral health, can lead to the presence of other bacteria that cause damage to the health of the individual, such as in aspiration pneumonia. The literature mentions that children with gastrostomy have significantly more plaques and calculus on their teeth, and more Haemophilus influenzae, with a tendency to more gram-negative, Pseudomonas, and Streptococcus pneumonia bacteria, leading to more aspiration pneumonia than children without gastrostomy^8^.

Finally, this study reinforced the understanding that children and young people with neurological impairment have changes in oral hygiene and that greater attention should be paid to oral hygiene care to prevent other complications in the individual's health. It should also be recognized that this study has limitations in relation to the analysis of oral hygiene, as the evaluation involved two aspects of plaques and calculations; perhaps the additional evaluation of caries, as well as other bacteria, would introduce more information that could be discussed. Thus, future studies are needed to clarify these data.

However, this study contributes to confirming the need for preventive oral hygiene guidelines and programs in this population. In addition, it highlights the need for interdisciplinary care involving physicians, speech therapists, nurses, dentists, and nutritionists, so that various comorbidities can be minimized in children and young people with neurological involvement and oropharyngeal dysphagia, consequently bringing gains in the quality of life for individuals and their families.

## Conclusion

Children and young people with neurological impairment and oropharyngeal dysphagia using gastrostomy had worse oral hygiene than who had no gastrostomy. There were no difference between the groups about bacteria type in the oral cavity, and both groups had just the bacterium *Streptococcus mutans.*
